# Supplementary material for: Hydrogen-bonds mediate liquid-liquid phase separation of mussel derived adhesive peptides
Source: Nat Commun. 2022 Oct 1;13:5771. doi: 10.1038/s41467-022-33545-w (PMC9526746; doi:10.1038/s41467-022-33545-w)
Supplement: Supplementary file 1 — Supplementary Information [file 41467_2022_33545_MOESM1_ESM.docx]

**Supplementary Information**

**Hydrogen-bonds mediate liquid-liquid phase separation of mussel derived adhesive peptides**

Qi Guo^1,#^, Guijin Zou^2,#^, Xuliang Qian^3^, Shujun Chen^1^, Huajian Gao^2,3,*^, Jing Yu^1,4*^

^1^ School of Materials Science and Engineering, Nanyang Technological University (NTU), 50 Nanyang Avenue, Singapore 637553, Singapore

^2^ Institute of High Performance Computing, A*STAR, Singapore 138632, Singapore

^3^ School of Mechanical and Aerospace Engineering, Nanyang Technological University (NTU), 50 Nanyang Avenue, Singapore 639798, Singapore

^4^ Institute for Digital Molecular Analytics and Science, Nanyang Technological University (NTU), 50 Nanyang Avenue, 637553, Singapore

^#^ These authors contributed equally to the study

^*^ Authors for correspondence: yujing@ntu.edu.sg; [huajian.gao@ntu.edu.sg](mailto:huajian.gao@ntu.edu.sg)

**Content**

Supplementary Figure 1: Full sequence of Mfp-5……………………………………..……………….4

Supplementary Table 1: Fitting CD data of GK-16* with Bestsel software………………………...…4

Supplementary Figure 2: Phase diagram of GK-16* with different anions………………………….....4

Supplementary Figure 3: FTIR spectra of GK-16*…………………………………………….…...….5

Supplementary Figure 4: CD spectra of GK-16 and GK-16*…………………………………………..5

Supplementary Table 2: Molecular mass of GK-16 derived peptides………….………………..……..6

Supplementary Figure 5: MALDI spectra of GK-16 derived peptides…………………………………7

Supplementary Figure 6: Zeta potential measurements of GK-16*…………………...……...………..8

Supplementary Figure 7: GK-16 before enzymatic modification shows no LLPS phenomenon……....8

Supplementary Figure 8: GK-16* shows gel-like or precipitate-like aggregates under PBS buffer…...9

Supplementary Figure 9: UV-Vis spectra of GK-16* at different pH……………………………….…9

Supplementary Figure 10: Phase diagram plotted of GK-16* at pH 7………………………..………10

Supplementary Figure 11: Representative configurations of peptides in MD simulations..……...…..10

Supplementary Figure 12: Optical microscopic images of GK-16 variant coacervates………………11

Supplementary Figure 13: Phase behavior of GK-16-2G……………………………………….…….11

Supplementary Figure 14: CD spectra of GK-16 derived peptides…………………………………...12

Supplementary Figure 15: Phase diagrams of GK-16-KK/GG*……………………………………….13

Supplementary Figure 16: Representative root-mean-square deviation of peptides in simulations……13

Supplementary Figure 17: The representative radius of gyration of peptides in simulations…………..14

Supplementary Figure 18: The root-mean-square fluctuations GK-16 derived peptides………………14

Supplementary Figure 19: Representative temporal evolutions of COM distances between the pairs..15

Supplementary Figure 20: Representative configurations of GK-16 variants in MD simulations……..16

Supplementary Figure 21: Representative configurations of GK-16* variants in MD simulations..….17

Supplementary Figure 22: The occupancy of most probable H-bond between different residues……..18

Supplementary Figure 23: Quantification of the number of H-bonds formed as a function of time…..18

Supplementary Figure 24: SFA experiments of GK-16*……………………………………….……..19

Supplementary Figure 25: Illustration of pH-dependent SFA experiments……………..…………….19

Supplementary Figure 26: SFA force-distance profiles of different GK-16 derived peptides………….20

Supplementary Table 3: Summary of adhesion forces (mN/m) measured by SFA…………..………..20

Supplementary Figure 27: SFA experiments of GK-16-GG/YY* and GK-16-2G*……………….…..21


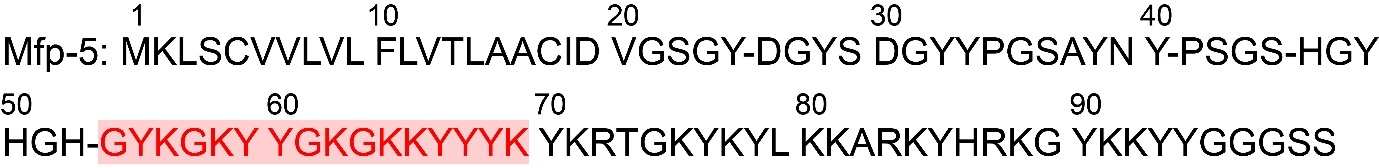


Supplementary Figure 1. Full sequence of Mfp-5. The highlighted region is the sequence of GK-16.

Supplementary Table 1. Fitting CD data of GK-16* with Bestsel software with ionic strength of 1 mM, 50 mM and 600 mM.

| Ionic strength (mM) | α-helix (%) | β-sheet (%) | β-turn (%) | Random coil (%) |
| --- | --- | --- | --- | --- |
| 1 | 23.8 | 0 | 0 | 76.2 |
| 50 | 6.4 | 36.4 | 14.3 | 42.8 |
| 600 | 0 | 53.9 | 6.3 | 39.8 |


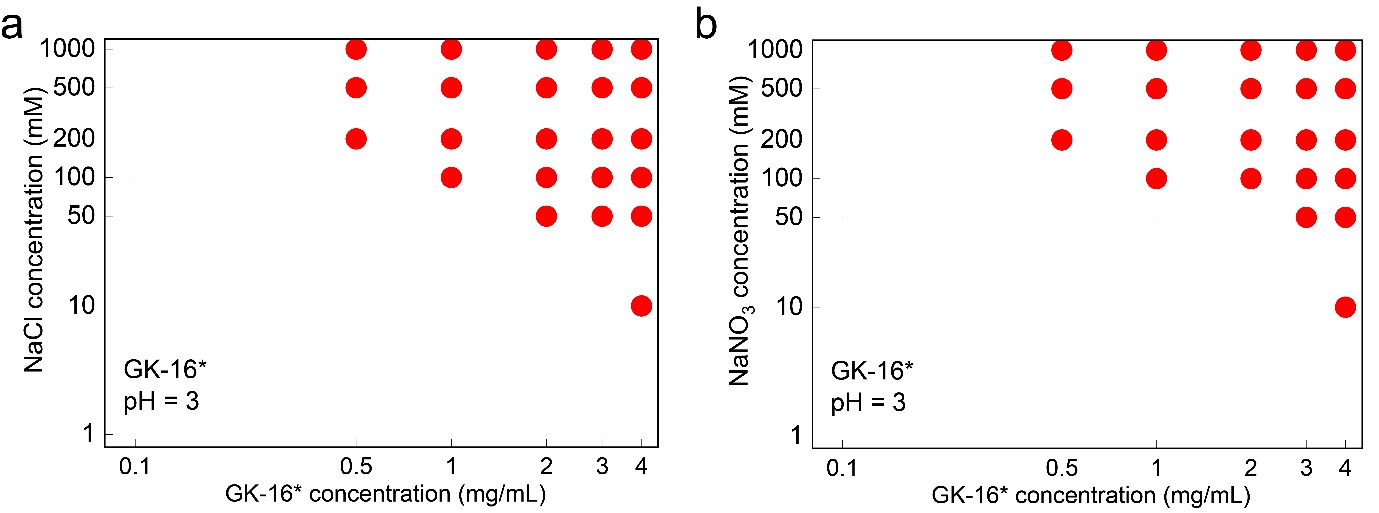


Supplementary Figure 2. Phase diagram of GK-16* with different anions. (a) NaCl; (b) NaNO_3_.

Supplementary Figure 3. FTIR spectra of GK-16* (5 mg/mL) in pH 3 DCl solution with ionic strength of 1 mM, 600 mM and 2000 mM and their difference spectra compared to the one measured with ionic strength of 1mM. All spectra are normalized to peak.


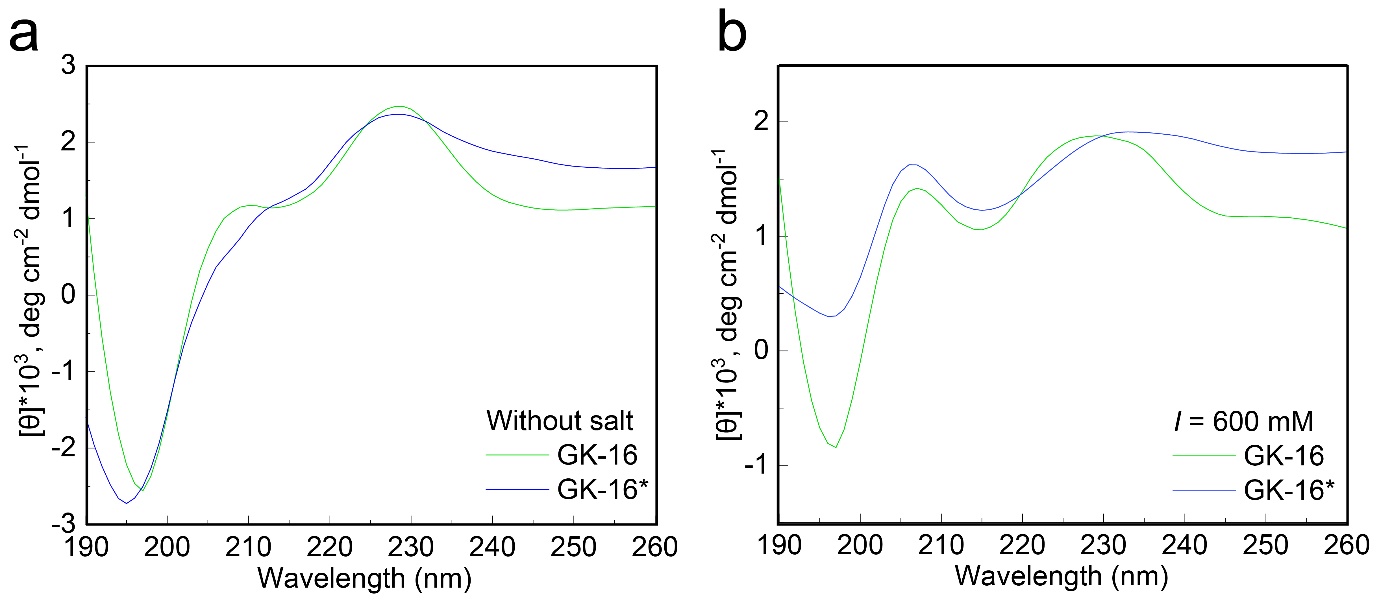


Supplementary Figure 4. CD spectra of GK-16 and GK-16* (1 mg/mL) in pH 3 HCl solution (a) without salt addition and (b) with ionic strength of 600 mM.

Supplementary Table 2. Molecular mass before and after enzymatic modification, predicted pI value, abbreviation and sequence of GK-16 derived peptides

| Abbreviation | Sequence | Mw | Mw  (of MALDI) | Mw  (modification) | Mw  (modification of MALDI) | pI |
| --- | --- | --- | --- | --- | --- | --- |
| GK-16 | GYKGKYYGKGKKYYYK | 1993 | 1994 | 2073 | 2074 | 9.85 |
| GK-16-GG/YY | YYKYKYYGKGKKYYYK | 2206 | 2207 | 2286 | 2287 | 9.75 |
| GK-16-G | GGKGKGGGKGKKGGGK | 1357 | 1358 | N/A | N/A | 10.70 |
| GK-16-1G | GGKGKYYGKGKKYYYK | 1887 | 1888 | 1951 | 1952 | 9.92 |
| GK-16-2G | GYKGKGGGKGKKYYYK | 1781 | 1782 | 1829 | 1830 | 10.00 |
| GK-16-3G | GYKGKYYGKGKKGGGK | 1675 | 1676 | 1723 | 1724 | 10.10 |
| GK-16-1G2G | GGKGKGGGKGKKYYYK | 1675 | 1676 | 1707 | 1708 | 10.10 |
| GK-16-1G3G | GGKGKYYGKGKKGGGK | 1569 | 1570 | 1601 | 1602 | 10.22 |
| GK-16-2G3G | GYKGKGGGKGKKGGGK | 1463 | 1464 | 1479 | 1480 | 10.40 |
| GK-16-2S3S | GYKGKSSGKGKKSSSK | 1612 | 1613 | 1628 | 1629 | 10.40 |
| GK-16-2A3A | GYKGKAAGKGKKAAAK | 1534 | 1535 | 1550 | 1551 | 10.40 |
| SK-16 | SYKSKSSSKSKKSSSK | 1732 | 1733 | 1748 | 1749 | 10.40 |
| GK-16-KK/GG | GYKGKYYGKGGGYYYK | 1582 | 1583 | 1932 | 1933 | 9.63 |


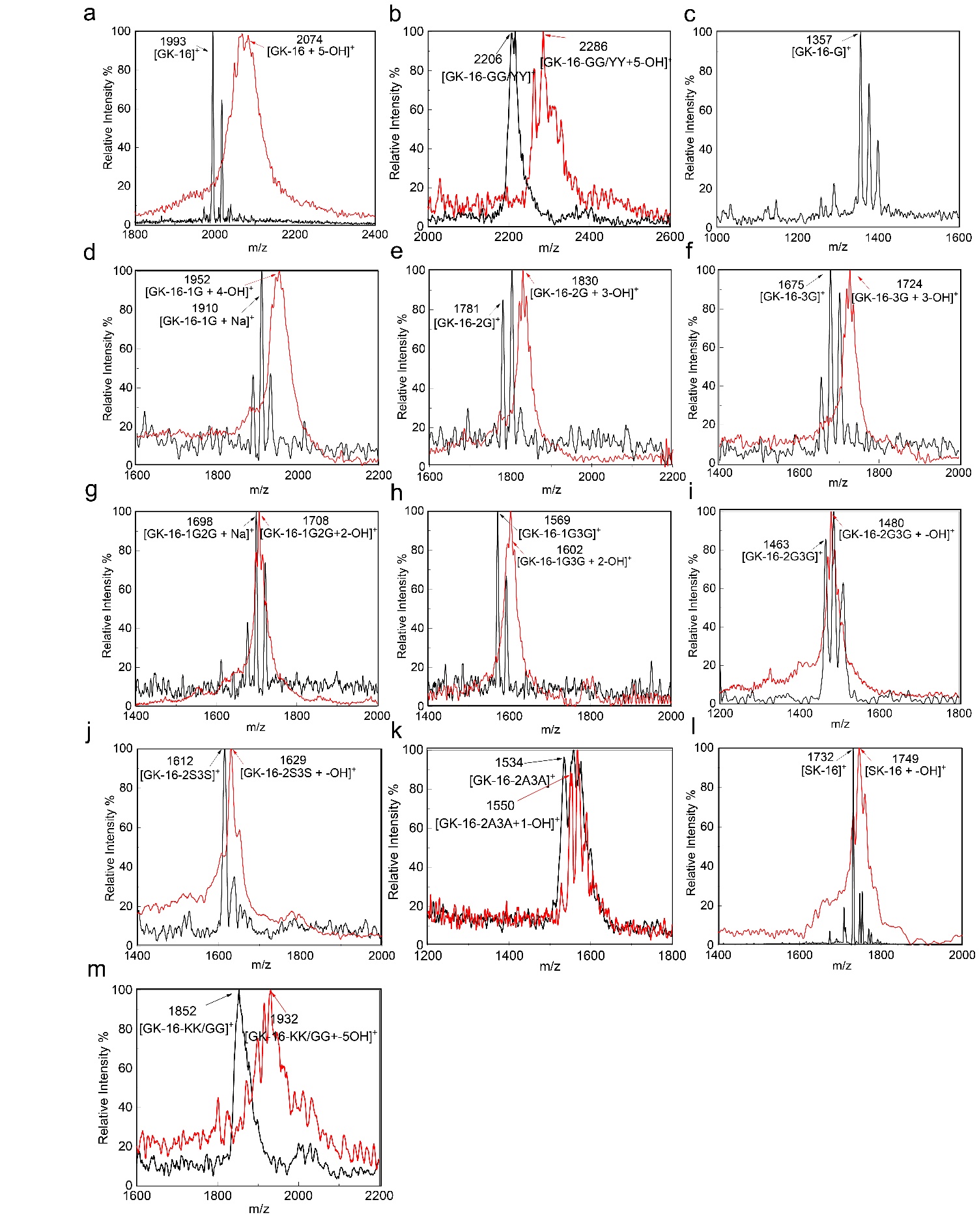


Supplementary Figure 5. MALDI spectra before and after enzymatic modification of (a) GK-16; (b) GK-16-GG/YY; (c) GK-16-G; (d) GK-16-1G; (e) GK-16-2G; (f) GK-16-3G; (g) GK-16-1G2G; (h) GK-16-1G3G; (i) GK-16-2G3G; (j) GK-16-2S3S; (k) GK-16-2A3A; (l) SK-16; (m) GK-16-KK/GG.


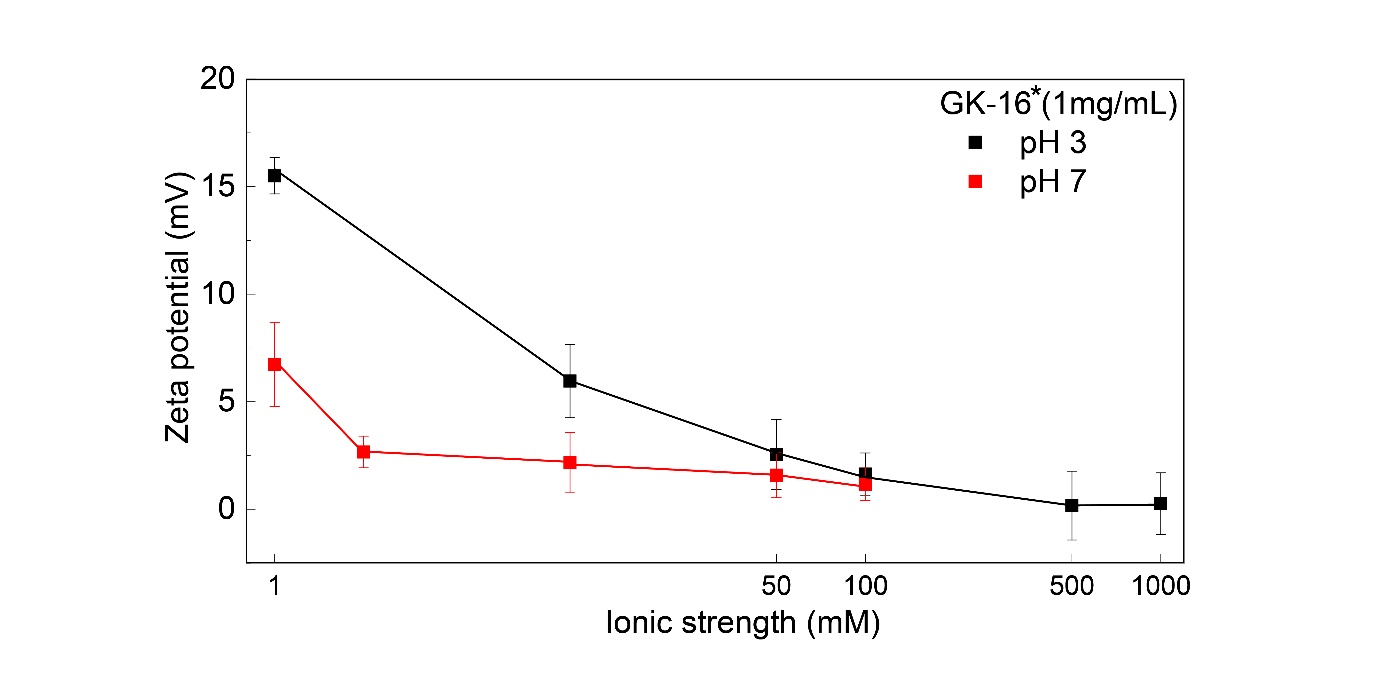


Supplementary Figure 6. Zeta potential measurements of GK-16* (1 mg/mL) under pH 3 (black curve) and pH 7 (red curve) with different ionic strength. *n* = 3 biologically independent samples over 3 independent experiments. Data are presented as mean values +/- SD.


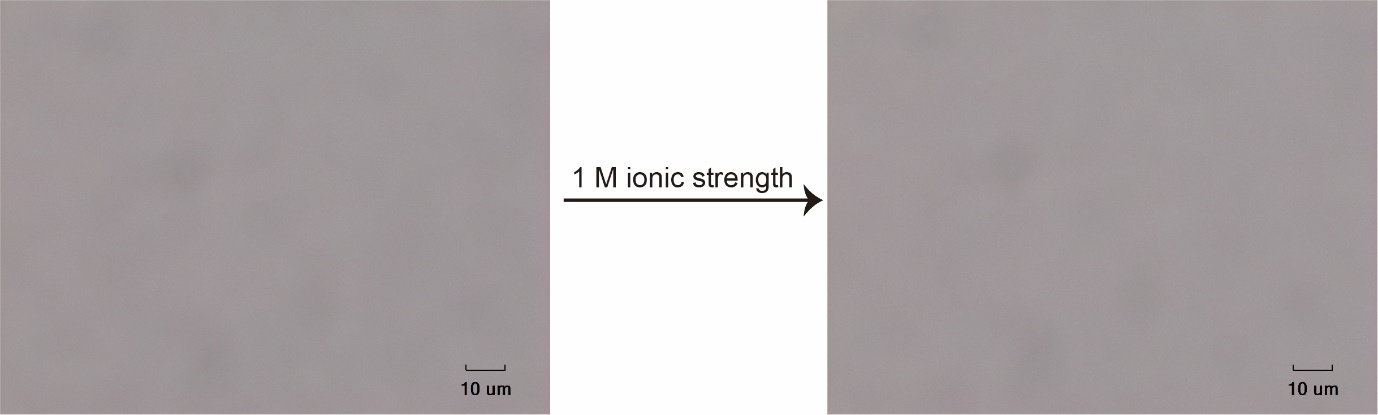


Supplementary Figure 7. GK-16 (2 mg/mL) before enzymatic modification shows no LLPS phenomenon under 1 M ionic strength. 3 experiments of each sample were repeated independently with similar results.


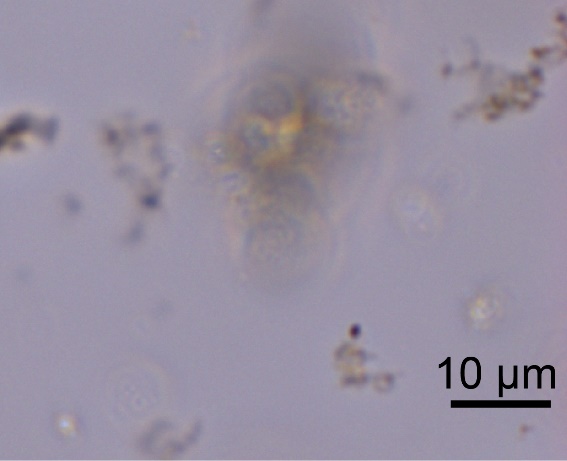


Supplementary Figure 8. GK-16* (2 mg/mL) shows gel-like or precipitate-like aggregates under PBS buffer (pH 7.4, ionic strength = 100 mM). 3 experiments of each sample were repeated independently with similar results.


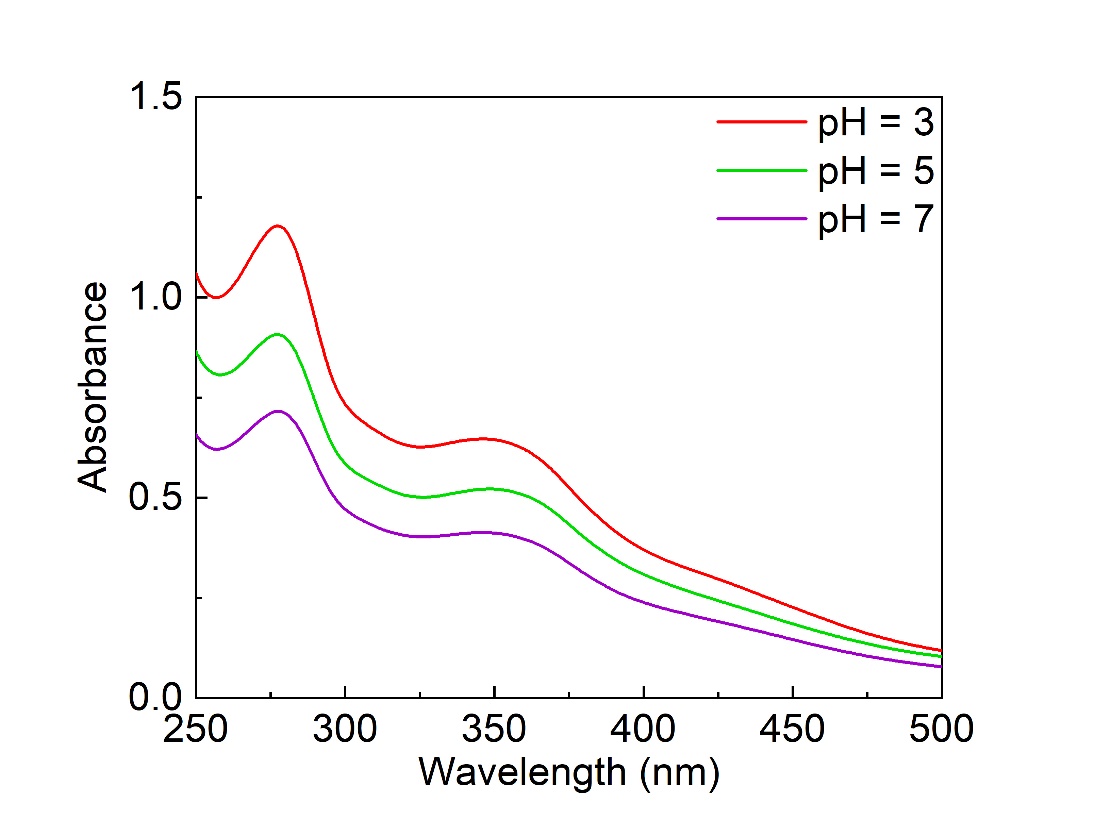


Supplementary Figure 9. UV-Vis spectra of GK-16* (1 mg/mL) under pH 3 (acetate buffer, 0.1 M), pH 5 (acetate buffer, 0.1 M) and pH 7 (PBS buffer, 0.1 M).

Supplementary Figure 10. Phase diagram plotted of GK-16* concentration vs urea concentration under 650 mM ionic strength, pH 7.4.


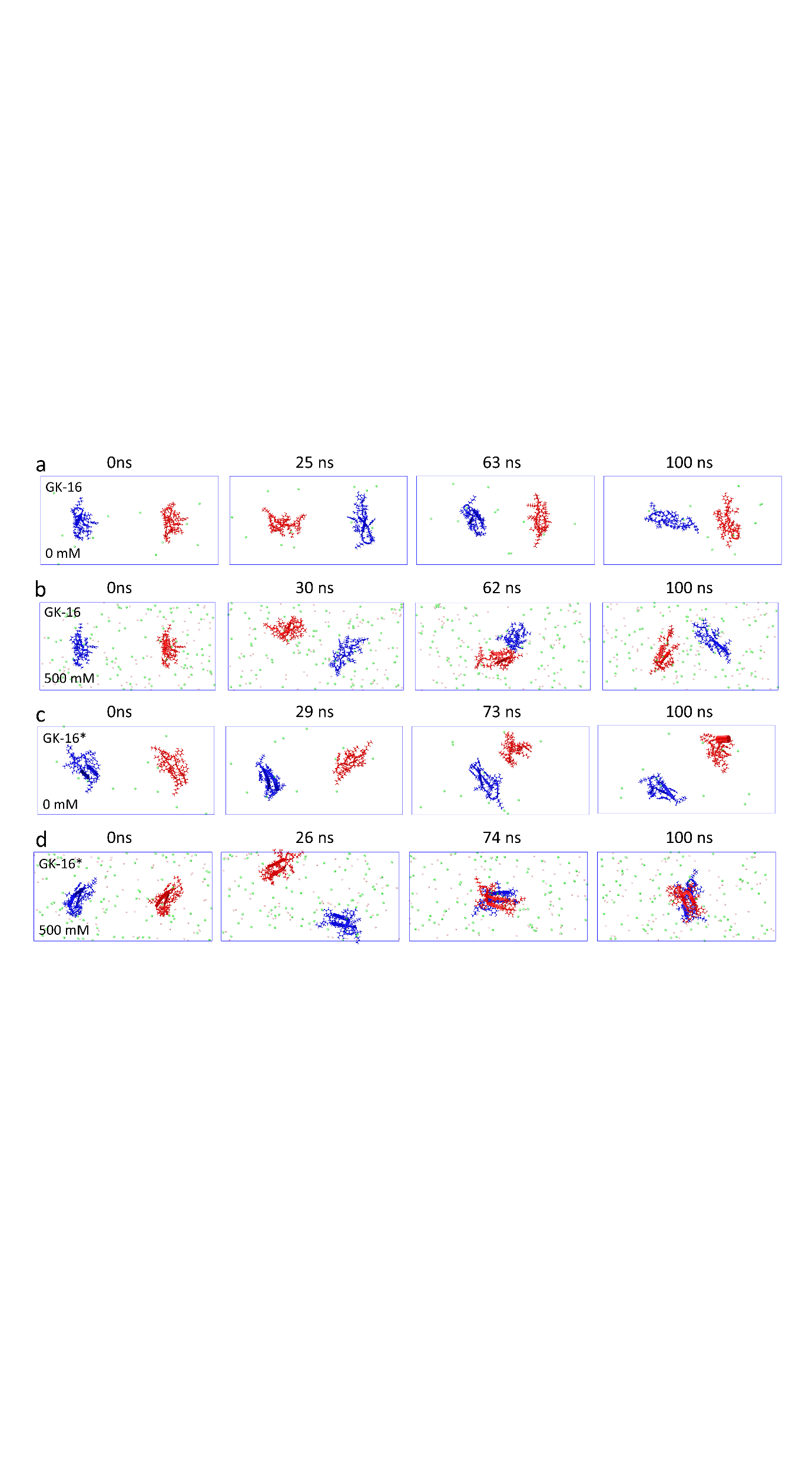


Supplementary Figure 11. Representative configurations of peptides in MD simulations. (a) GK-16 under 0 mM; (b) GK-16 under 500 mM; (c) GK-16* under 0 mM; (d) GK-16* under 500 mM; Sodium chloride are depicted as spheres and colored as follows: chloride: green; sodium pink. Water molecules are not shown for clarity.


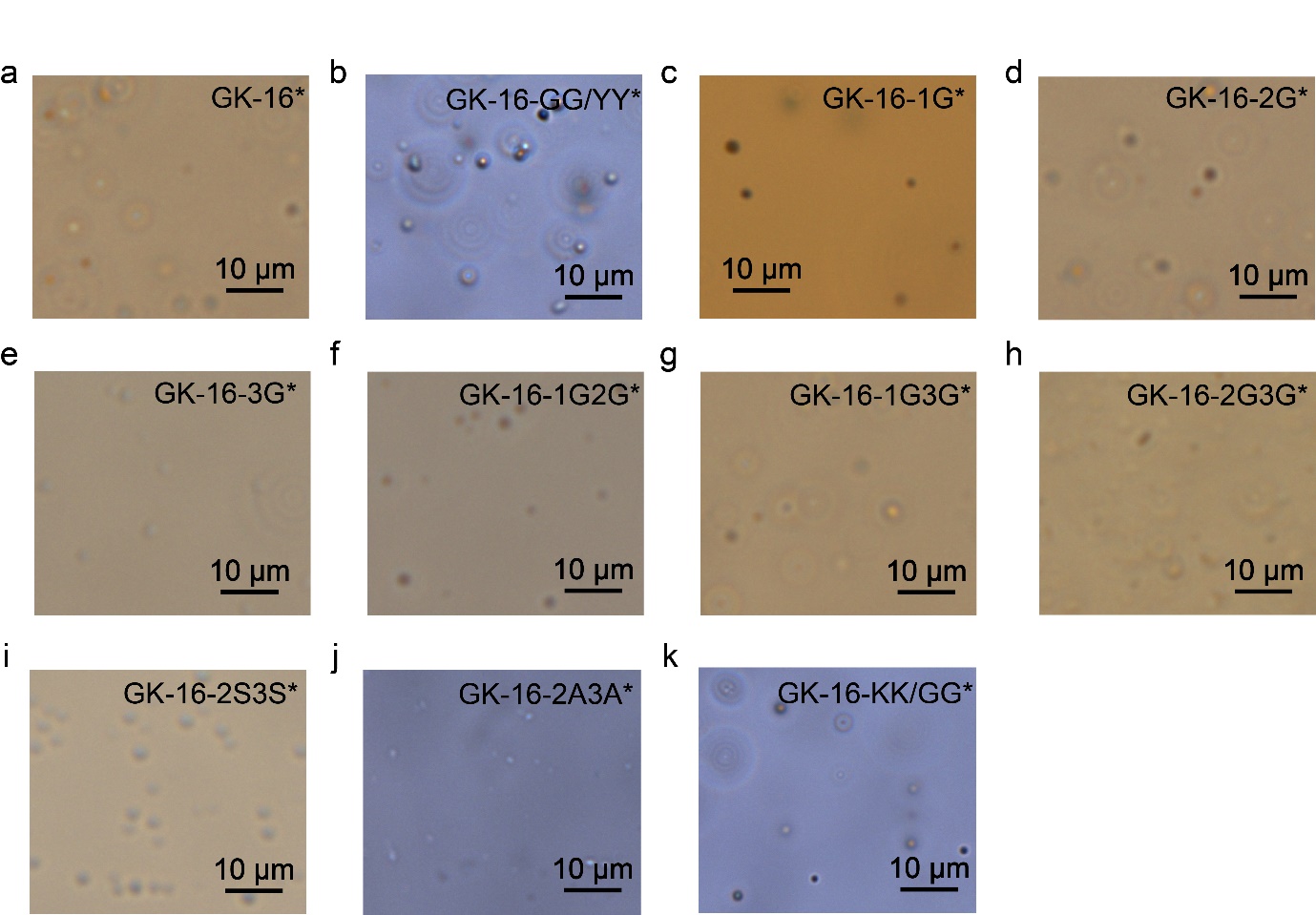


Supplementary Figure 12. Optical microscopic images of GK-16 variant (2 mg/mL) coacervates. (a) GK-16*; (b) GK-16-GG/YY*; (c) GK-16-1G*; (d) GK-16-2G*; (e) GK-16-3G*; (f) GK-16-1G2G*; (g) GK-16-1G3G*; (h) GK-16-2G3G*; (i) GK-16-2S3S*; (j) GK-16-2A3A*; (k) GK-16-KK/GG*. 3 experiments of each sample were repeated independently with similar results.


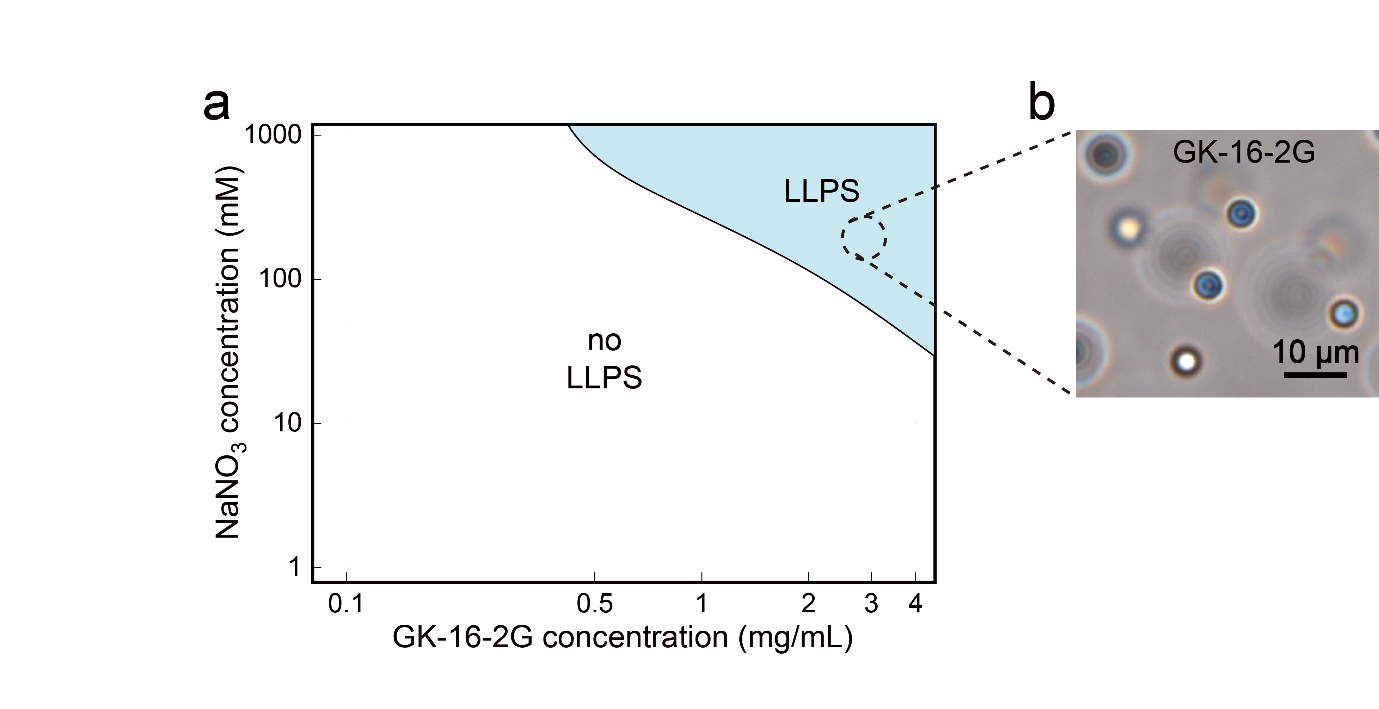


Supplementary Figure 13. Phase behavior of GK-16-2G. (a) Phase diagram of GK-16-2G with different peptide and salt concentrations at pH 3. (b) By adding salt (*I* = 1 M), GK-16-2G (2 mg/mL) forms coacervates. 3 experiments were repeated independently with similar results.


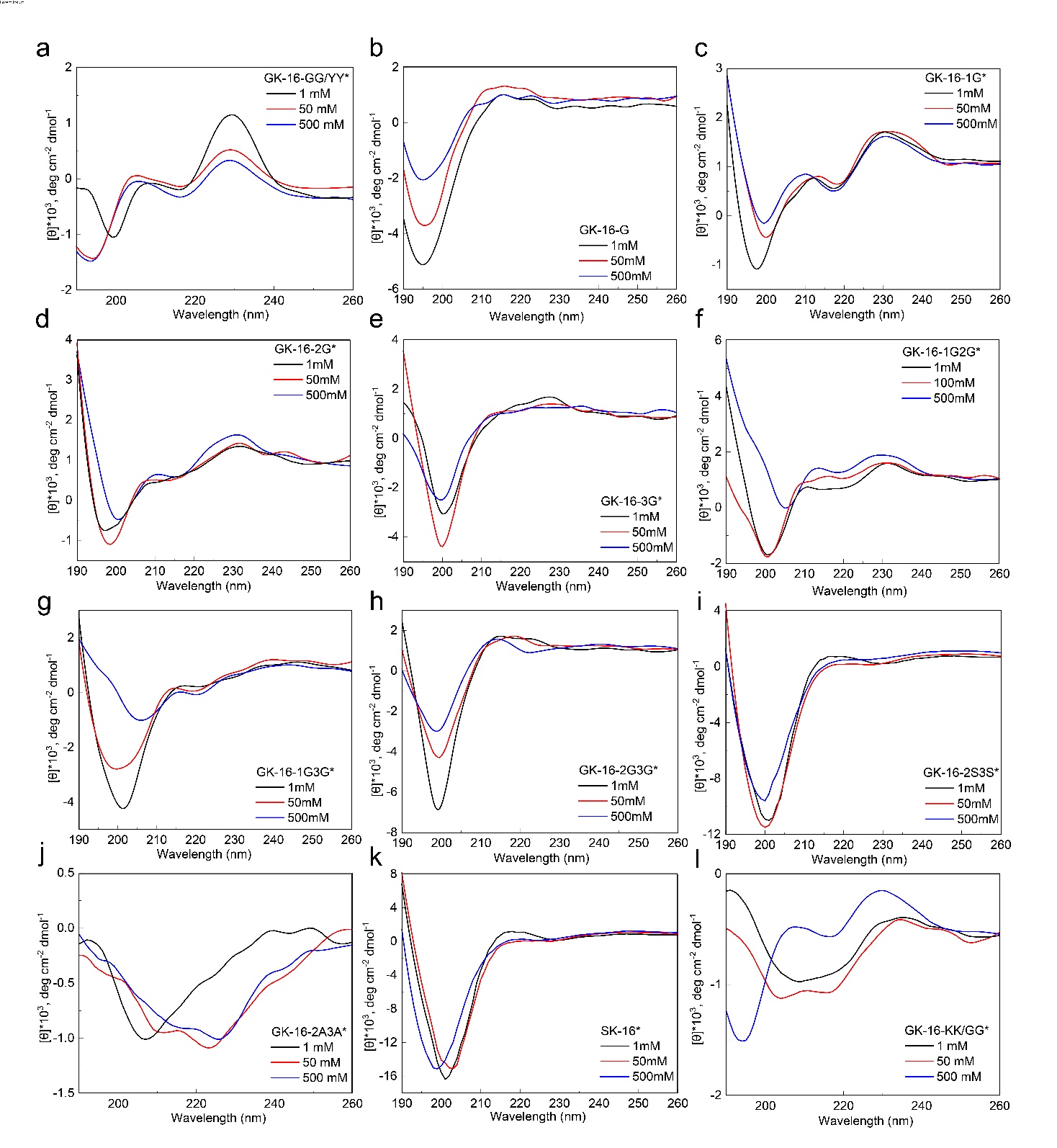


Supplementary Figure 14. CD spectra of GK-16 derived peptides (1 mg/mL). (a) GK-16-GG/YY*; (b) GK-16-G; (c) GK-16-1G*; (d) GK-16-2G*; (e) GK-16-3G*; (f) GK-16-1G2G*; (g) GK-16-1G3G*; (h) GK-16-2G3G*; (i) GK-16-2S3S*; (j) GK-16-2A3A*; (k) SK-16*; (l) GK-16-KK/GG*.


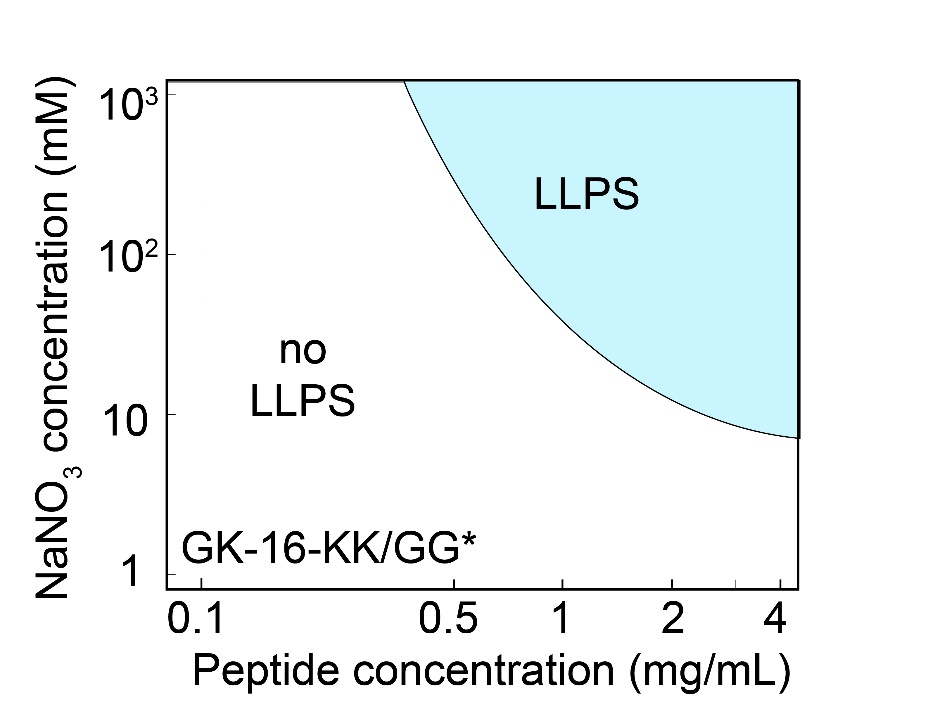


Supplementary Figure 15. Phase diagrams of GK-16-KK/GG*.





Supplementary Figure 16. Representative root-mean-square deviation (RMSD) of peptides in simulations.





Supplementary Figure 17. The representative radius of gyration of peptides in simulations.





Supplementary Figure 18. The root-mean-square fluctuations (RMSFs) of (a) the original GK-16 and (b) the Dopa modified GK-16* peptides.





Supplementary Figure 19. The representative temporal evolutions of COM distances between the pairs of GK-16 variants as well as their Dopa-modified conversions in MD simulations.


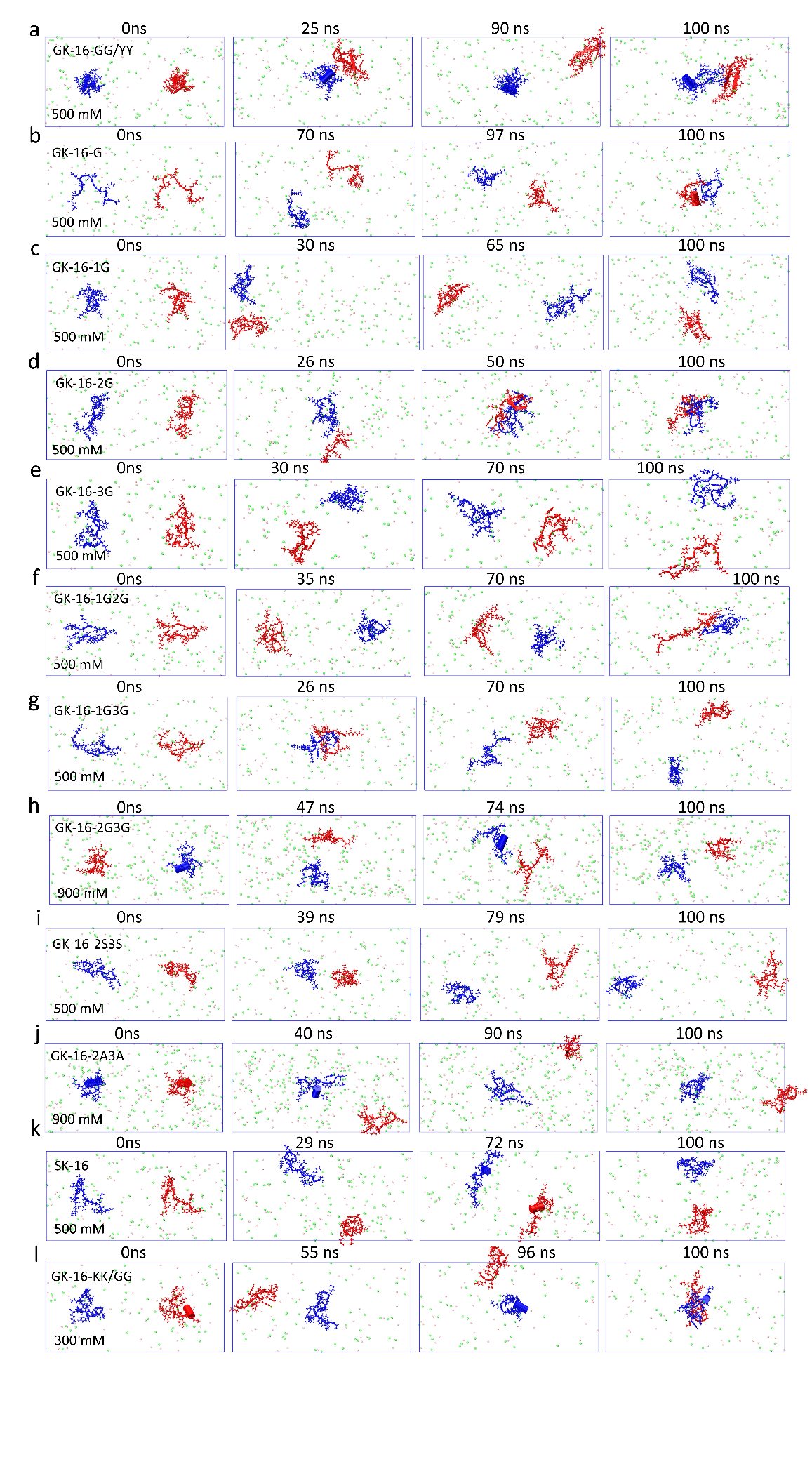


Supplementary Figure 20. The representative configurations of GK-16 variants in MD simulations. (a) GK-16-GG/YY; (b) GK-16-G; (c) GK-16-1G; (d) GK-16-2G; (e) GK-16-3G; (f) GK-16-1G2G; (g) GK-16-1G3G; (h) GK-16-2G3G; (i) GK-16-2S3S; (j) GK-16-2A3A; (k) SK-16; (l) GK-16-KK/GG.


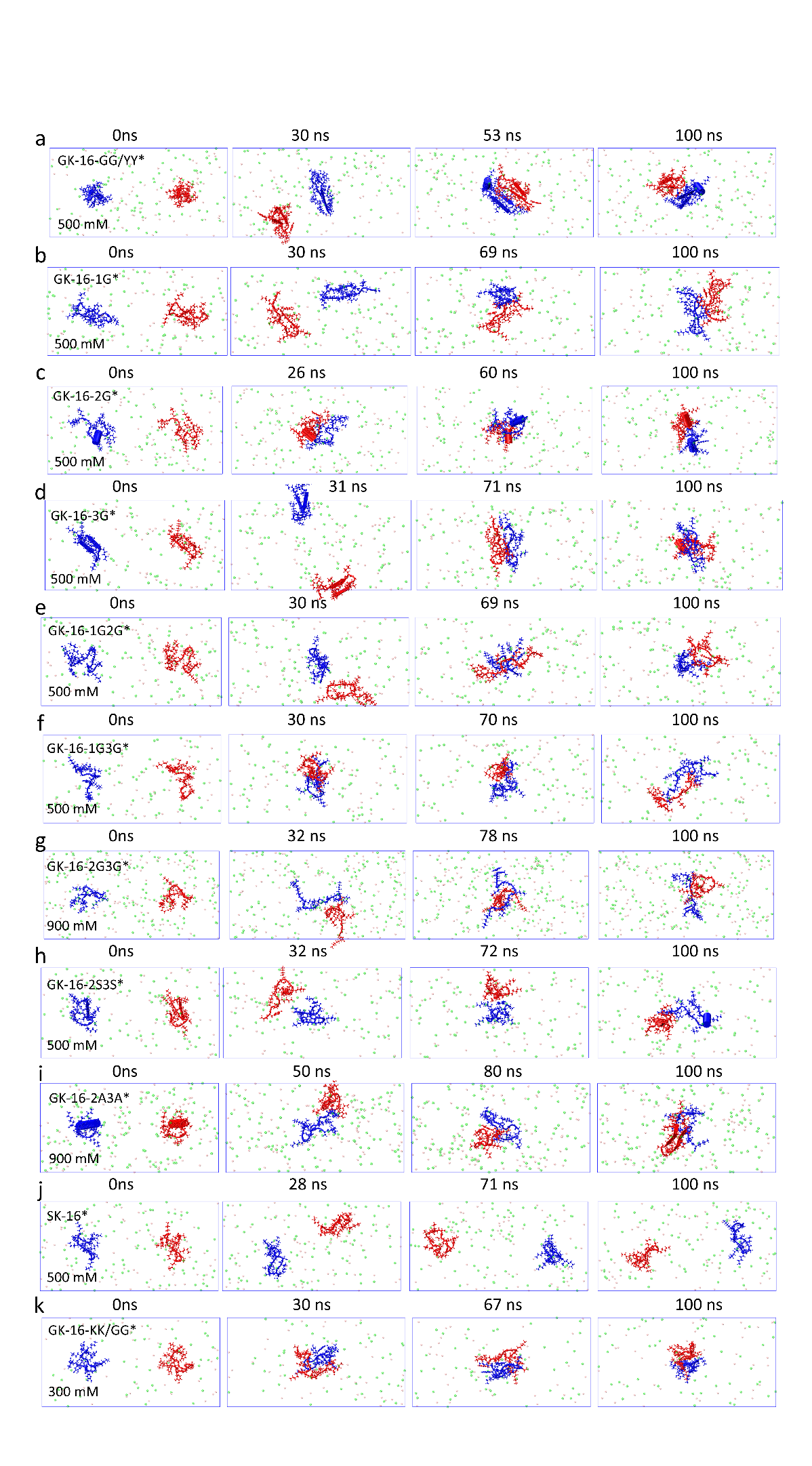


Supplementary Figure 21. The representative configurations of Dopa-modified GK-16 variants in MD simulations. (a) GK-16-GG/YY*; (b) GK-16-1G*; (c) GK-16-2G*; (d) GK-16-3G*; (e) GK-16-1G2G*; (f) GK-16-1G3G*; (g) GK-16-2G3G*; (h) GK-16-2S3S*; (i) GK-16-2A3A*; (j) SK-16*; (k) GK-16-KK/GG*.


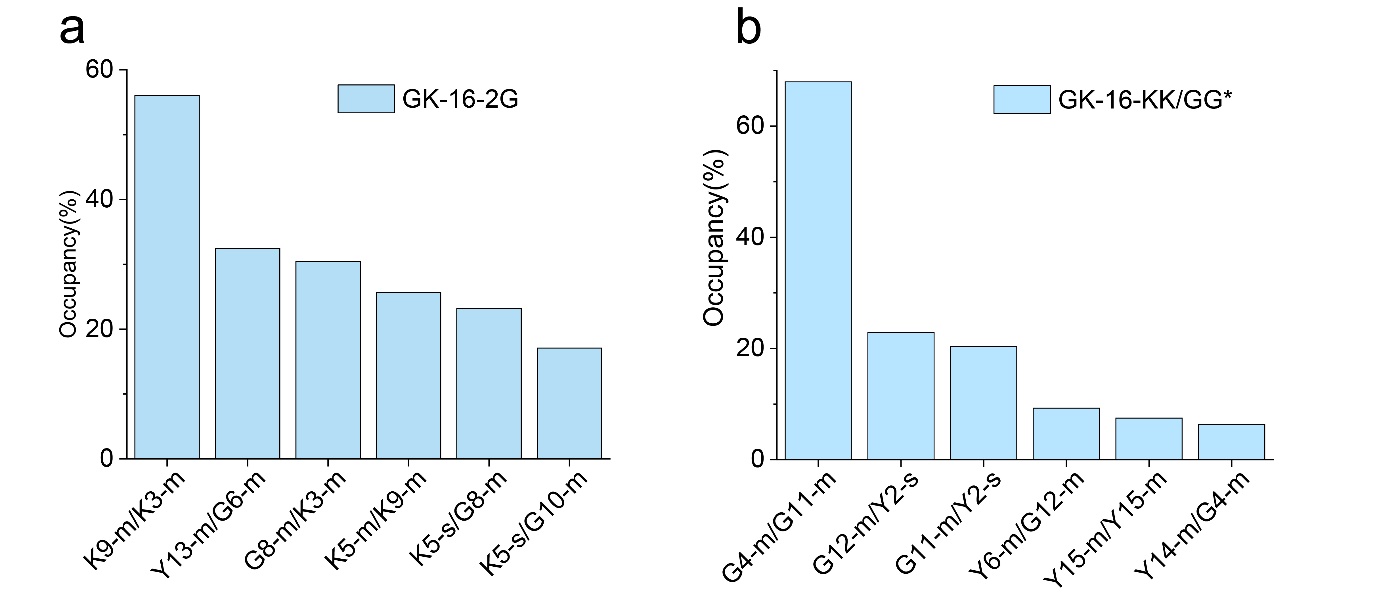


Supplementary Figure 22. The occupancy of most probable H-bond between different residues was obtained from the last 40 ns of simulations of (a) GK-16-2G; (b) GK-16-KK/GG*.





Supplementary Figure 23. Quantification of the number of H-bonds formed as a function of time during the 100 ns MD simulation between different pairs of corresponding peptides.


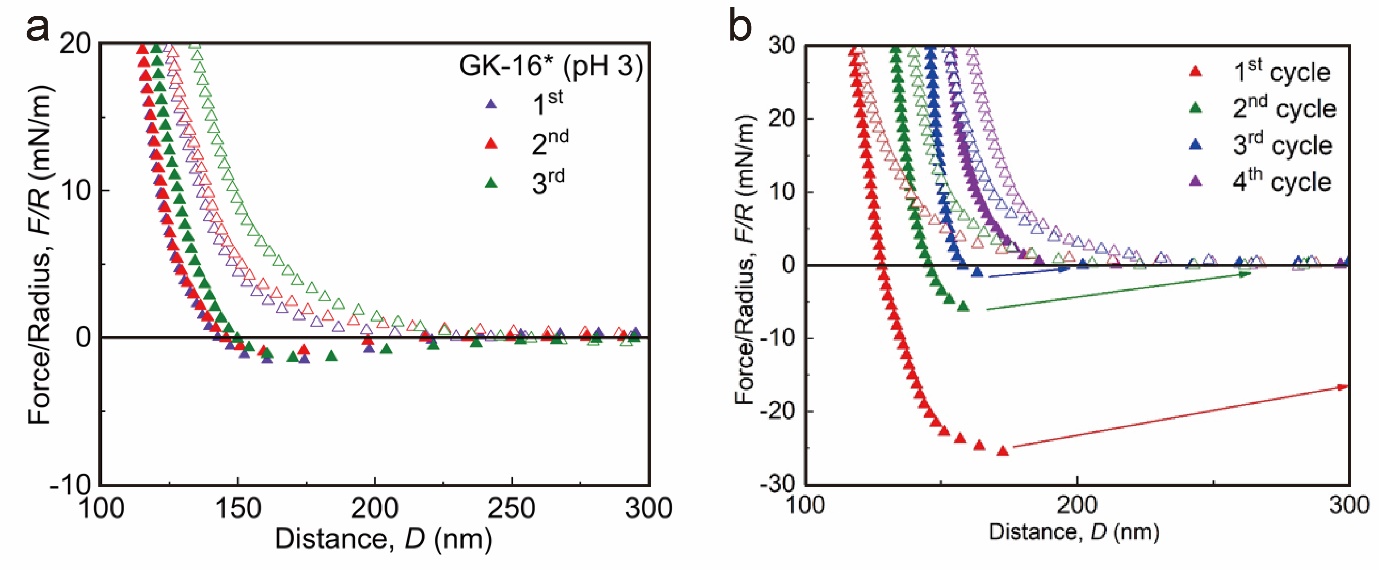


Supplementary Figure 24. SFA experiments of GK-16*. (a) SFA force-distance profiles of GK-16* (2 mg/mL) in HCl solution (pH 3, 0.1 M ionic strength with 200 mM urea) showed reproducible and consistent adhesion force. (b) SFA force-distance profiles of GK-16* (2 mg/mL) in PBS buffer (0.1 M, pH = 7.4) showed that the adhesion force rapidly decreased to zero after 4 cycles.


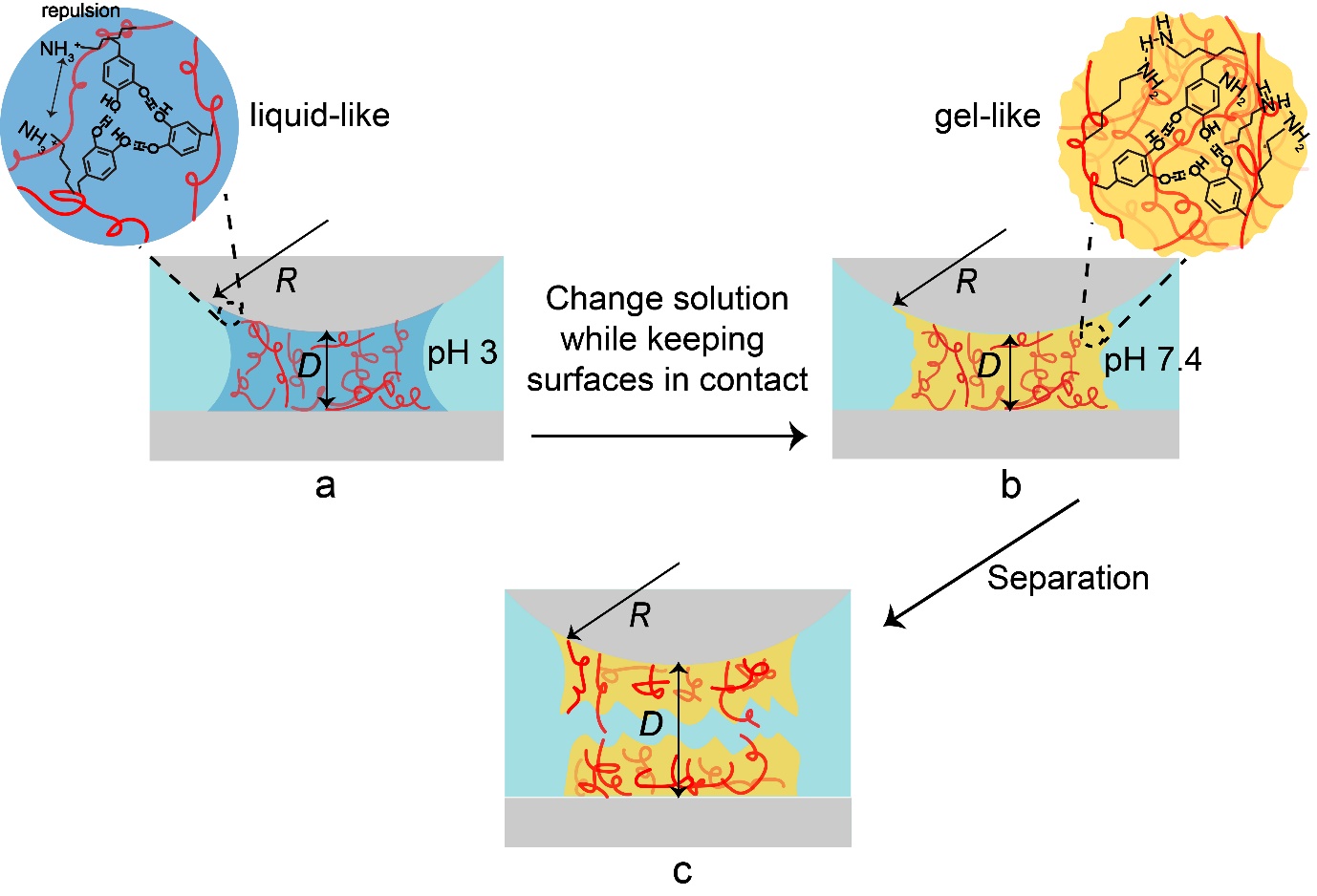


Supplementary Figure 25. Illustration of pH-dependent SFA experiments. (a) Peptides form liquid-like condensates at pH 3; (b) After keeping surfaces in contact and changing pH to 7.4, complexes become more gel-like. (c) Upon separation, the cohesive failure happens with a large adhesion force.


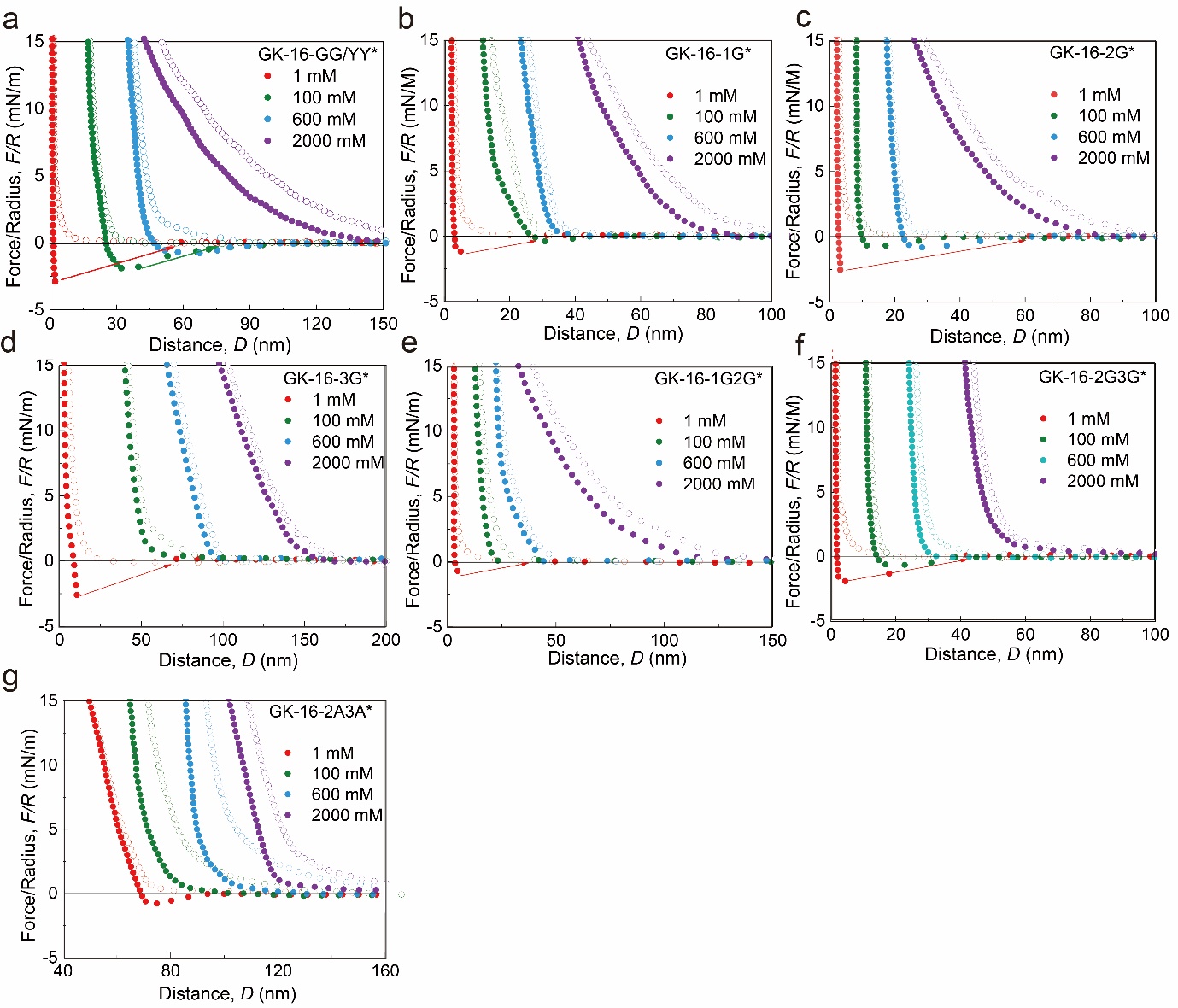


Supplementary Figure 26. SFA force-distance profiles of different GK-16 derived peptides (2 mg/mL) in solutions with different ionic strengths. (a) GK-16-GG/YY*; (b) GK-16-1G*; (c) GK-16-2G*; (d) GK-16-3G*; (e) GK-16-1G2G*; (f) GK-16-2G3G*; (g) GK-16-2A3A*.

Supplementary Table 3. Summary of adhesion forces (mN/m) measured by SFA for different GK-16 derived peptides (2 mg/mL) with different ionic strength.

| Abbreviation | 1 mM | 100 mM | 600 mM | 2000 mM |
| --- | --- | --- | --- | --- |
| GK-16* | -1.05±0.08 | -1.24±0.13 | -1.34±0.14 | N/A |
| GK-16-GG/YY* | -1.73±0.24 | -1.60±0.41 | -0.74±0.06 | N/A |
| GK-16-1G* | -1.22±0.08 | N/A | N/A | N/A |
| GK-16-2G* | -2.92±0.58 | -0.66±0.09 | -0.85±0.25 | N/A |
| GK-16-3G* | -2.68±0.24 | N/A | N/A | N/A |
| GK-16-1G2G* | -0.64±0.09 | N/A | N/A | N/A |
| GK-16-2G3G* | -1.84±0.28 | -0.59±0.06 | N/A | N/A |
| GK-16-2A3A* | -0.72±0.14 | N/A | N/A | N/A |


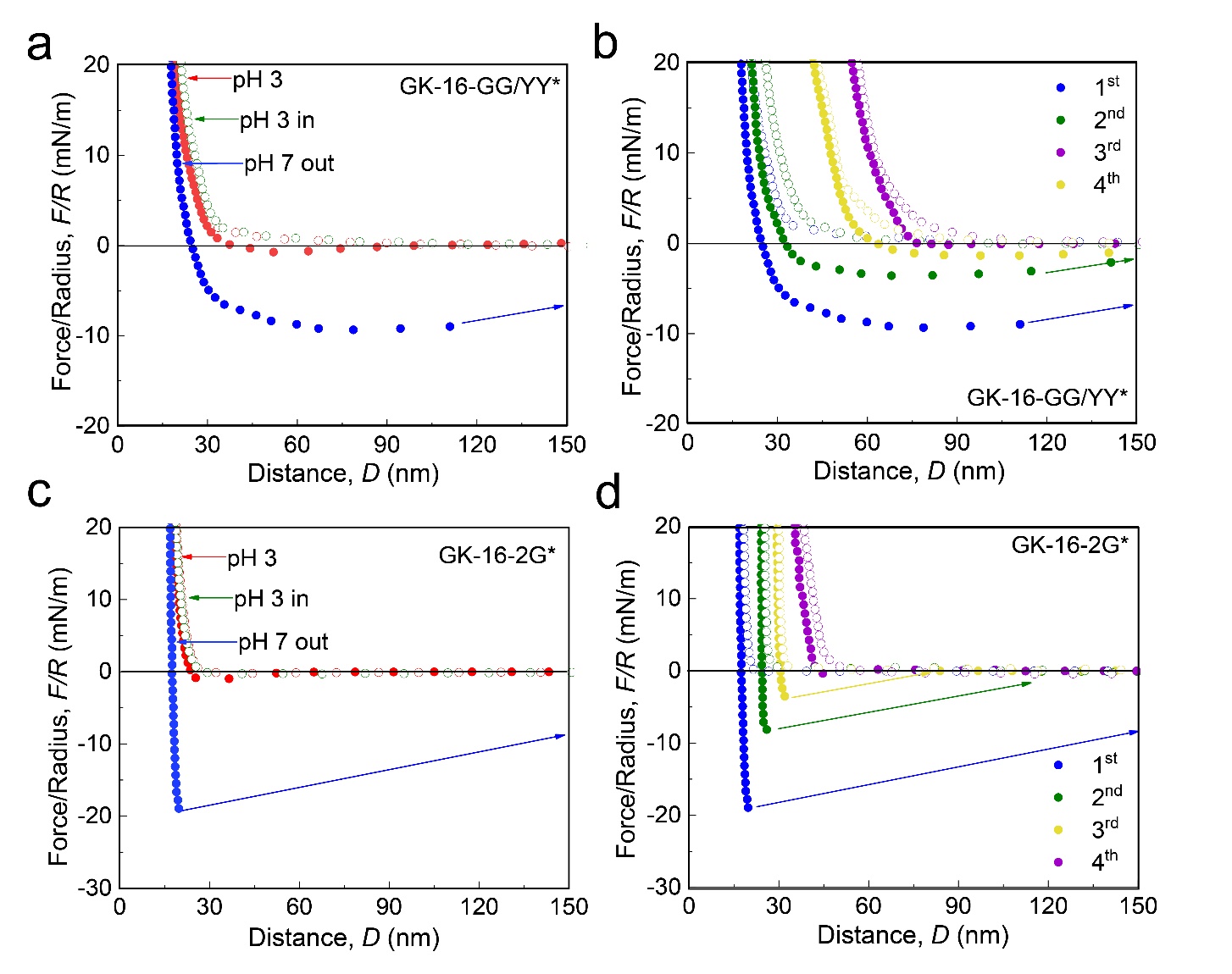


Supplementary Figure 27. SFA experiments of GK-16-GG/YY* and GK-16-2G*. SFA measurements on (a) GK-16-GG/YY* coacervate and (c) GK-16-2G* (2 mg/mL, 100 mM ionic strength) indicate a liquid-to-gel transition by changing the solution pH from 3 to 7.4. SFA force-distance profiles of (b) GK-16-GG/YY* and (d) GK-16-2G* (2 mg/mL) in PBS buffer (0.1 M, pH = 7.4) showed that the adhesion force rapidly decreased to zero after 4 cycles.
